# Corticolimbic Structural Deficits in Violent Patients with Schizophrenia

**DOI:** 10.3390/brainsci15030224

**Published:** 2025-02-21

**Authors:** Maria Athanassiou, Alexandre Dumais, Inès Zouaoui, Alexandra Fortier, Luigi de Benedictis, Olivier Lipp, Andràs Tikàsz, Stéphane Potvin

**Affiliations:** 1 Centre de Recherche de l’Institut Universitaire en Santé Mentale de Montréal, Montreal, QC H1N 3V2, Canada; maria.athanassiou@umontreal.ca (M.A.); alexandre.dumais@umontreal.ca (A.D.); ines.zouaoui@umontreal.ca (I.Z.); luigi.de.benedictis@umontreal.ca (L.d.B.); olivier.lipp.med@ssss.gouv.qc.ca (O.L.); andras.tikasz@mail.mcgill.ca (A.T.); 2 Department of Psychiatry, Faculty of Medicine, University of Montreal, Montreal, QC H3T 1J4, Canada; 3 Institut National de Psychiatrie Légale Philippe-Pinel, Montreal, QC H1C 1H1, Canada

**Keywords:** schizophrenia, sMRI, violence, emotion, amygdala

## Abstract

**Background/Objectives**: Violent behaviors are uncommon in patients with schizophrenia (Sch), but when present, exacerbate stigma and challenge treatment. The following study aimed to identify the structural abnormalities associated with violent behaviors in Sch by implementing a validated tool specifically designed to evaluate violent behaviors in psychiatric populations, as well as by performing region-of-interest neuroimaging analyses, focused on areas commonly associated with the neurobiology of violence and aggression. **Methods**: Eighty-three participants were divided into three groups: Sch with violent behaviors (Sch+V, *n* = 34), Sch without violent behaviors (Sch-V, *n* = 28), and healthy controls (HC, *n* = 21). Structural neuroimaging analyses were performed across groups to assess gray matter volume (GMV) and cortical thickness (CT) differences in regions previously implicated in aggressive behaviors. **Results**: The data revealed significant reductions in GMV in the right amygdala and diminished cortical thickness (CT) in the bilateral dorsolateral prefrontal cortices (dlPFC) in patients with Sch+V compared to patients with Sch-V and HCs. Right amygdalar volume also demonstrated a negative correlational trend with hostility scores in patients with Sch+V. **Conclusions**: These findings underscore disruptions in the structural integrity of the dlPFC—responsible for inhibitory control—and the amygdala—central to emotional processing in violent patients with Sch. Future research should aim to investigate potential functional interactions at a network level to gain a deeper understanding of the neurobiological underpinnings of violent behaviors in this population.

## 1. Introduction

Most individuals affected by Sch disorders do not exhibit aggressive or violent behaviors [1,2]. Yet, a significant association between schizophrenia and hostile behaviors has been established [3,4]. Key factors linked to violence in this population include male gender [5,6], symptomatology (e.g., higher positive symptoms, lack of insight) [7,8,9], treatment non-adherence [10], substance use [3], and comorbid personality disorders [11]. Despite the prevalence of Sch hovering around 1% globally [12], individuals with schizophrenia account for over 6% of homicides [13]; importantly, only a minority of patients affected by Sch commit most of the violence [14]. The perpetration of interpersonal violence associated with these diagnoses has profound implications for victims, families, and caregivers, significantly increasing their burden [15]. Violent behaviors also reinforce stigma against individuals with schizophrenia and interfere with treatment efforts [4,10,16,17]. Considering the high clinical importance of aggression in Sch, there has been growing interest in investigating the neural signatures of violence in this patient population.

Structural and functional magnetic resonance imaging (MRI) studies in non-psychotic populations with aggressive or violent behaviors have identified differences in brain regions involved in cognitive control, emotion regulation, and reward processing. A synthesis of nine studies involving aggression-prone individuals showed increased limbic (amygdala and hippocampus) and temporal activity with reduced occipital activity, supporting the limbic hyperactivity model in anger-eliciting contexts [18]. In addition, a meta-analysis of 83 functional MRI studies involving antisocial individuals showed reduced activations in the anterior cingulate cortex (ACC) and (anterior) insula and dorsolateral prefrontal cortex (dlPFC) during threat perception tasks; increased activity in the striatum, medial PFC, and dlPFC during social cognition tasks; as well as decreased activity in the (anterior) insula and dlPFC during a cognitive control task [19]. Structural MRI studies have produced results coherent with the functional findings, revealing volumetric reductions in youth and adults exhibiting aggressive or antisocial behaviors in executive prefrontal regions, such as the dlPFC and ventrolateral PFC (vlPFC); reduced volumes in limbic regions like the amygdala and insula—which play central roles in emotional processing—and abnormal brain volumes in reward-related regions, namely the ventromedial PFC and striatum [20,21,22,23]. Although less attention has been paid to the types of aggressive behavior, preliminary structural neuroimaging evidence suggests that amygdala volumetric abnormalities may underlie reactive aggression, which stems from emotion regulation difficulties [24]—while goal-directed proactive aggression has been associated with increased gray matter volumes in the (ventral) striatum [22].

The neurobiological bases of aggressive or antisocial behaviors in individuals with Sch have been investigated in a relatively small number of neuroimaging studies. Using tasks assessing executive functions, a few functional magnetic resonance imaging (fMRI) studies have shown that these behaviors are associated with reduced activity in the dlPFC and the orbitofrontal cortex (OFC) [25,26]. Using emotional tasks, neural alterations have been observed in the ACC, the dlPFC, and the vlPFC [27,28,29]. A systematic review based on a limited number of studies has highlighted that the ACC seems the most often impaired brain region in this population, regardless of the task used in the scanner [30]. A few functional connectivity studies have shown that aggressive or antisocial behaviors are associated with impaired coupling between the ACC and other prefrontal regions (dlPFC and lateral OFC) [31,32], as well as between the amygdala and prefrontal regions (ACC, medial OFC and vlPFC) [33]. Although results are preliminary, structural neuroimaging studies have also identified structural brain alterations associated with violent/aggressive or antisocial behaviors in Sch populations, mostly volumetric differences in fronto-limbic regions and cortical thickness alterations in the superior temporal and the fusiform cortices [34,35,36,37,38,39]. Systematic reviews of this literature have shown that aggressive or antisocial behaviors are associated with GMV reductions, particularly in the dlPFC, vlPFC, (medial) OFC, ACC, insula, and hippocampus, as well as abnormal volumes in the amygdala and striatum in Sch [40,41,42,43]. However, these reviews all noticed that study samples were often small and that results were quite heterogeneous across studies. Methodological variability, including differences in sample characteristics, definitions and measures of aggression (e.g., verbal and/or physical; minor vs. severe; self-report vs. interview), and neuroimaging approaches (e.g., region-of-interest vs. whole-brain analyses), have likely contributed to the lack of consensus in the literature [40]. Recently, the schizophrenia working group from the ENIGMA consortium conducted a cross-sectional study across 20 international sites, including 2095 patients with schizophrenia. The authors determined that aggressive behaviors were significantly associated with reductions in GMV in the dlPFC and the inferior parietal lobule [44]. Although this study included the largest number of aggressive patients with Sch to date, aggressive behaviors were assessed using the Hostility item from the Positive and Negative Syndrome Scale (PANSS) [45], which measures irritability rather than aggressive behaviors per se.

The following study aims to extend our current knowledge in the field by identifying the distinct structural abnormalities associated with violent behaviors in schizophrenia spectrum disorders, using a validated instrument specifically designed to assess violent behaviors in psychiatric populations, and implementing region-of-interest analyses based on seeds commonly implicated in the neurobiology of violence and aggression. Considering the literature in both psychotic and nonpsychotic populations, as well as based on our own prior work investigating functional connectivity alterations in the violent schizophrenia population [31], we postulate that structural alterations in limbic areas (such as the amygdala) and prefrontal regions (such as the dlPFC) will be significantly associated with violent antecedents in individuals with schizophrenia.

## 2. Materials and Methods

### 2.1. Participants

Participants consisted of 83 adults aged 18 to 60 years. They were divided into three groups: schizophrenia with violent behavior (Sch+V; N = 34), schizophrenia without violent behavior (Sch-V; N = 28), and healthy controls (HC; N = 21). Patients with Sch were primarily recruited from the Institut Universitaire en Santé Mentale de Montréal, while HCs were recruited from the general Montreal community. Diagnoses of schizophrenia-spectrum disorders were established using the psychotic, mood, and substance disorders modules from the Structured Interview for DSM-5 criteria [46]. Medical records from the psychiatric facility were also consulted. Violent behavior was defined by a score of at least 1 on any of the first five questions of the MacArthur Community Violence Instrument (MACVI), which indicates a history of violent behavior such as stabbing, shooting, serious injury requiring or not requiring hospitalization, and murder [47,48]. Sch+V had a score of 1 or more on the MACVI, whereas patients with Sch-V all had a score of 0. The presence or absence of a history of violent behavior was verified by consulting medical records.

Exclusion criteria for all participants included (1) any acute, unstable, or uncontrolled physical illness; (2) neurological disorders; (3) a substance use disorder in the past 12 months; and (4) any contraindications to undergoing an MRI scan (e.g., pregnancy, metal implants, claustrophobia). HCs were further excluded if they had (1) a psychiatric disorder as per DSM-5 criteria or (2) were currently taking any medications affecting the central nervous system. Patients with Sch unable to provide informed consent were also excluded. All participants gave written informed consent in accordance with the Declaration of Helsinki, and the study was approved by the local research ethics committee.

### 2.2. Clinical Assessments

Sociodemographic data were collected, including age, sex, and handedness. Severity of positive, negative, and general symptoms in patients with Sch was measured using the Positive and Negative Syndrome Scale (PANSS) [45]. Aggressive and violent behaviors, as well as their frequency, were assessed using the MacArthur Community Violence Instrument (MACVI) [47]. The MACVI was selected as it had been validated for psychiatric populations [49,50], making it a relevant instrument for our population of interest; also, it has been implemented in previous neuroimaging studies in the Sch+V population, including studies from our team [28,31,51]. Focusing on the first 5 questions from the MACVI allowed us to center our attention on the more severe forms of violent behaviors, i.e., forms of behaviors which would cause life-threatening harm or death to other persons. The SCID-II was administered for the purpose of determining the number of diagnostic criteria for conduct (15 items) and antisocial personality (7 items) disorders that were met by patients with Sch+V and Sch-V [52]. Antipsychotic dosage was calculated using chlorpromazine equivalents [53]. None of the patients with Sch were antipsychotic-naïve.

### 2.3. Neuroimaging Acquisition Parameters

Images were acquired at the Unité de Neuroimagerie Fonctionnelle de l’Institut de Gériatrie de l’Université de Montréal on a Siemens Prisma Fit 3.0 Teslas scanner. High-resolution T1-weighted anatomical images were acquired using the following parameters: TR = 2300 ms; TE = 2.98 ms; FA = 9°; matrix size = 256 × 256; voxel size = 1 mm^3^; with 176 sagittal slices.

### 2.4. Image Preprocessing and Gray Matter Volume Analysis

T1-weighted images were processed using the FreeSurfer software package (version 6.0.0) via the CBRAIN platform [54]. The standard processing pipeline included intensity normalization, skull-stripping, tissue segmentation, and parcellation based on anatomical atlases. The primary outcomes were the volumes of gray matter structures previously identified in the pathophysiology of violent behaviors in individuals with and without psychosis, including the left and right amygdala and the left and right striatum (caudate, putamen, and nucleus accumbens), and cortical thickness in regions such as the medial and lateral OFC, the right and pars orbitalis of the inferior frontal gyrus (i.e., vlPFC), caudal middle frontal gyrus (i.e., dlPFC), caudal ACC, and insula. These regions of interest (ROIs) were a priori defined and based on the most consistent results from previous (functional and structural) neuroimaging meta-analyses on violent behavior in non-psychotic individuals, as well as the growing neuroimaging literature on violent behavior in Sch [40,43,44]. No whole-brain analyses were performed. Total intracranial volume (TIV) was calculated and included as a covariate in all statistical models.

### 2.5. Statistical Analyses

All statistical analyses were performed in R v4.1.3, using the packages stats, car, effectsize, emmeans, and rstatix. Group differences in demographic and clinical variables were assessed using ANOVAs or Student’s *t*-tests for continuous variables (if variables met the assumptions of normality and homoscedasticity) and χ^2^ tests for categorical variables. To identify potential differences between groups in gray matter volume (GMV) and cortical thickness (CT), we performed multivariate analysis of covariance (MANCOVA) models. The first model included the GMV of the right and left amygdala and the right and left striatum as dependent variables, with groups (Sch+V, Sch-V, HC) as independent variables and estimated TIV as a covariate. The second model included CT of the bilateral medial orbitofrontal, the left and right lateral orbitofrontal, the left and right pars orbitalis, the left and right caudal middle frontal, the bilateral caudal anterior cingulate, and the left and right insula as the dependent variables, with the same independent variable and covariate as in the first model. For both models, the dependent variables were continuous, linear, normally distributed, and had equal variance–covariance matrices across groups. In addition, the dependent and covariate variables were linear, and the three groups had parallel lines with homoscedasticity (no significant interaction between the independent variable and the covariate), indicating equal slopes and variances meeting the additional assumptions required for MANCOVA models [55]. Additional models were created with chlorpromazine equivalents and clozapine status as covariates. However, the outcomes of these models did not significantly differ from the original models, and as such, the original models were retained as final results. Effect sizes were calculated (partial η^2^ at the model level and Cohen’s d at the post hoc level). False discovery rate (FDR) was used to control for type I errors that may arise from multiple comparisons in the analyses on GMV (for the first model) and CT (for the second model) in each set of hypothesis tests with multiple comparisons [56,57]. Finally, Pearson’s correlations were performed to examine potential associations between structural alterations and antipsychotic dosage, antisocial personality traits, and psychiatric symptoms, including the P7 item (Hostility) from the PANSS. There were no missing data for either MANCOVA model. However, some clinical data were missing in a minority of participants, so we excluded these participants from the correlation analyses.

## 3. Results

### 3.1. Participant Demographics

Patients with schizophrenia in the Sch+V and Sch-V sub-groups both had long-duration illness (14.4 years and 14.7 years, respectively). Patients with Sch+V had histories of one or more of the following behaviors: murder (*n* = 6), inflicted injury requiring hospitalization (*n* = 16), inflicted firearm injury (*n* = 2), stabbing (*n* = 9), and hurting a person with any other object (*n* = 17). The mean number of lifetime violent behaviors was 3.3 ± 0.5. Healthy controls (HCs), participants with nonviolent schizophrenia (Sch-V), and participants with violent schizophrenia (Sch+V) did not differ significantly in terms of age, sex, or handedness. In addition, Sch-V and Sch+V participants did not significantly differ in terms of diagnosis (Sch vs. schizoaffective disorder), age of onset, duration of illness, chlorpromazine equivalents, or clozapine ratio (Table 1).

### 3.2. Gray Matter Volume (GMV) Analyses

**Figure 1:**
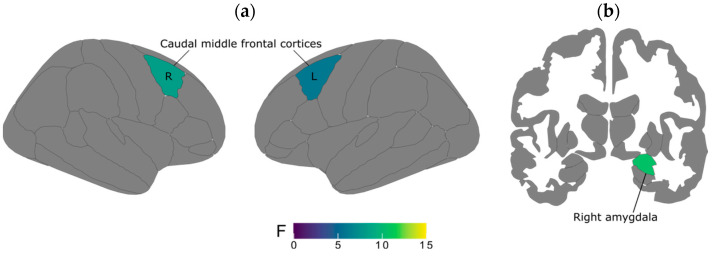
Graphical representation of regions of interest significantly altered in structural neuroimaging analyses in patients with Sch+V in (**a**) the bilateral dlPFC and (**b**) the right amygdala.

MANCOVA analyses for gray matter volume (GMV) revealed a significant group effect (Wilks’s Λ = 0.74, F = 3.01, *p* = 0.003) after adjusting for TIV. Significant between-group differences were observed in the right amygdala (Table 2; Figure 1). Specifically, the GMV of the right amygdala was significantly smaller in the Sch+V group compared to both HC and Sch-V groups (F = 10.63, *p* = 0.0003). Post hoc tests revealed a significant difference between HC vs. Sch+V (*p* = 0.0003; Cohen’s d = 0.93) and between Sch-V vs. Sch+V (*p* = 0.01; Cohen’s d = 0.62). No other significant group differences were found in the left amygdala or striatal regions (putamen, caudate and accumbens). Volumetric differences between patients with Sch+V and Sch-V in the right amygdala remained significant when clozapine ratio and chlorpromazine equivalents were considered as (separate) covariates. No correlation was observed between right amygdala volumes and chlorpromazine equivalents and PANSS-positive and -negative symptoms and antisocial personality traits across Sch groups (all *p*s > 0.05). However, we found a negative association between right amygdala volumes and hostility as measured with the P7 item (Hostility) from the PANSS in patients with Sch+V (r = −0.39; *p* = 0.04).

### 3.3. Cortical Thickness (CT) Analyses

MANCOVA analyses for cortical thickness (CT) revealed no significant group effect in specific regions (Wilks’s Λ = 0.66, F = 1.54, *p* = 0.08) after adjusting for TIV. It highlighted significant differences in the bilateral medial orbitofrontal (F = 4.96, *p* = 0.03) and caudal middle frontal cortices (left: F = 5.94, *p* = 0.02; right: F = 8.25, *p* = 0.006). With respect to the medial orbitofrontal cortex, Sch+V participants displayed thinner cortices compared to HCs (*p* = 0.03, Cohen’s d = 0.54), with no significant differences between Sch-V and Sch+V.

Concerning the right caudal middle frontal cortex (i.e., dlPFC), post hoc tests determined a significant difference between Sch+V and HCs (*p* < 0.001, Cohen’s d = 0.86), as well as Sch+V vs. Sch-V (*p* = 0.04; Cohen’s d = 0.52) (Table 3; Figure 1). Similar effects were also exhibited in the left caudal middle frontal cortex, whereby Sch+V showed reduced cortical thickness compared to Sch-V (*p* = 0.03, Cohen’s d = 0.53) and HCs (*p* = 0.008, Cohen’s d = 0.70). No other group differences were observed in the lateral OFC, the inferior frontal gyrus (pars orbitalis), the caudal ACC, and the insula. CT differences between patients with Sch+V and Sch-V in the bilateral dlPFC remained significant when the clozapine ratio and chlorpromazine equivalents were considered as (separate) covariates.

No correlation was observed between the (bilateral) dlPFC and chlorpromazine equivalents and PANSS-positive and -negative symptoms and antisocial personality traits across Sch groups (all *p*s > 0.05).

## 4. Discussion

In view of the limited and uncertain evidence on the structural deficits associated with antecedents of violence in patients with Sch, this paper performed neuroimaging morphometry analyses to examine the structural deficits in this complex population. When compared to healthy controls, violent patients with Sch exhibited a pattern of reduced GMV in the right amygdala, as well as lower cortical thickness in the bilateral dlPFC, compared to healthy controls and nonviolent patients with Sch.

Our findings of cortical thinning in the dlPFC align with prior works in violent or aggressive patients with Sch, particularly with the most recent bodies of structural neuroimaging evidence with the largest sample sizes [44] and studies using similar methodological protocols [58]. Structural abnormalities in the prefrontal regions, such as the dlPFC, have been linked to aggression, as these areas are considered key hubs for controlling top–down “brake” responses and are included in various neural models of human aggression [59,60]. Previous studies in non-psychotic populations have suggested that the dlPFC plays a key role in attenuating aggression in social contexts by regulating emotional responses [59,61]. For example, increased dlPFC activity after experiences of social rejection has been associated with reduced behavioral aggression [62,63]. Additionally, stronger functional connectivity between the lateral prefrontal cortex and limbic regions has been linked to decreased retaliatory aggression [64]. As such, we posit that structural deficits in the dlPFC in patients with schizophrenia are involved in the dysregulation of emotional experiences, whereby acts of violence would be inappropriately deployed to alleviate negative emotions, such as anger, fear, and frustration [65,66].

With respect to our findings of reduced GMV in the amygdala, there is evidence supporting its involvement in violent behaviors in non-psychotic populations [67,68]. Although preliminary evidence suggests that amygdala volumes are reduced in patients with Sch [68], such alterations have not been consistently identified in this specific population [41]. Nonetheless, our results are consistent with earlier seminal fMRI work derived from individuals with Sch, which identified decreased connectivity between the ventral prefrontal regions and the amygdala, which was inversely associated with aggressive behaviors [33]. Notably, these findings complement meta-analytic evidence of limbic hyperactivation, including the amygdala, in response to emotionally arousing stimuli in aggression-prone individuals without psychosis [18]. The role of the amygdala in emotional responding has been extensively investigated and established [69], including the lateralization of specific processes between the left and right amygdala [70]. The left amygdala appears to be more involved in detailed and sustained processing, especially for tasks involving conscious evaluation or language-related emotional cues; in contrast, emotionally arousing stimuli automatically engage the right amygdala and initiate a broad generalized emotional reaction [71], thought to be crucial for the rapid detection of dynamic emotional cues [72,73]. This lateralized function may underpin reactive aggression, an immediate and impulsive reaction towards a perceived threat [74], which has been linked to right amygdalar morphometric alterations, including lower volume and surface deformations in conditions like intermittent explosive disorder [75,76]. Interestingly, in the current study, we found a negative association between right amygdala volumes and hostility levels in patients with Sch+V. Taken together, these findings resonate with the notion that violence in Sch is predominantly reactive, meaning that it is driven by emotional dysregulation, rather than the planned, goal-directed nature of instrumental violence [77,78].

Overall, our findings provide evidence of significant structural deficits in the right amygdala and bilateral dlPFC in violent patients with Sch, which are brain regions integral to emotional responding, emotion regulation, and top–down cognitive control. Reduced amygdalar volume supports the postulated link between (reactive) aggression and heightened emotional reactivity, while dlPFC thinning aligns with impaired inhibitory control over aggressive impulses, as highlighted in prior functional and structural MRI studies in Sch [33,44]. The co-occurrence of these abnormalities underscores a disrupted interplay between limbic and prefrontal regions, which are key circuits involved in balancing emotional impulses with rational decision-making. These results expand on the literature by linking specific structural deficits to violence in Sch, emphasizing the need to further explore their functional interactions at a network level to better understand aggression in this population. In non-psychotic individuals, preliminary positron emission tomography (PET) studies have linked impulsiveness and aggressiveness to cortical and subcortical alterations in the serotonin transporter and the 5-HT2A receptor, as well as reduced monoamine oxidase-A density in the brain reward system [79]. PET studies would need to be conducted in patients with Sch+V to determine if similar alterations are found in this complex population.

## 5. Limitations

Our findings should be interpreted with consideration of the following limitations. First, the sample size, while sufficient to detect group differences of moderate-to-large magnitude, may have been too small to capture group differences of smaller magnitude. Second, antipsychotic medications exert complex effects on brain structure and function, which may have influenced our results [80]. While short-term treatment can normalize prefrontal activity [81], long-term use is linked to reductions in global brain and temporal lobe volumes [82]. Nonetheless, we found no significant correlations between chlorpromazine equivalents and the structural measures analyzed. Third, the predominance of male participants in our sample limits the applicability of our findings to female Sch populations, as structural differences between sexes have been documented in this disorder [83]. Although we used both categorical (e.g., MACVI) and dimensional (e.g., PANSS-P7 -Hostility and antisocial personality traits) approaches in the current study, our clinical assessment of violent behavior was not fully comprehensive and did not incorporate multiple risk factors. In particular, the absence of a measure distinguishing reactive from instrumental violence reduces the specificity of our findings. Although not mutually exclusive, these subtypes of violence are considered to involve distinct neural mechanisms. Future studies should address this limitation to better elucidate their respective neurobiological underpinnings in schizophrenia.

## 6. Conclusions

The current study aimed to examine the structural brain alterations associated with violent behaviors in patients affected by Sch. Our findings suggest that violent behaviors in Sch spectrum disorders are associated with reduced cortical thickness in the bilateral dlPFC, as well as reduced GMV in the right amygdala. Our results contribute to the expanding body of literature that underscores the pivotal role of brain regions involved in emotion regulation in violent behaviors in Sch. Future studies should evaluate the applicability of our findings to female Sch populations, incorporate larger sample sizes, and include measures that distinguish between reactive and instrumental violence to provide a more nuanced understanding of the neurobiological mechanisms underlying aggression in schizophrenia.

## Figures and Tables

**Table 1:** Summary of sociodemographic characteristics of participants.

	Sch-V (*n* = 34)	Sch+V (*n* = 28)	HC (*n* = 20)	Statistics
Age, mean (SE)	33.9 (1.3)	33.9 (1.7)	30.6 (1.8)	F = 1.26, *p* = 0.29
Sex, % male	91.2	96.4	85.0	χ^2^ = 1.96, *df* = 2, *p* = 0.38
Handedness, % right/% left	88.2/2.9	89.3/7.1	75.0/20.0	χ^2^ = 5.47, *df* = 4, *p* = 0.24
PANSS-positive, mean (SE)	16.4 (0.9)	17.0 (1.1)	-	T = −0.39, *p* = 0.70
PANSS-negative, mean (SE)	16.1 (1.2)	17.0 (1.3)	-	T = −0.50, *p* = 0.62
PANSS-generalized, mean (SE)	31.8 (1.1)	34.0 (2.1)	-	T = 0.93, *p* = 0.34
PANSS-total, mean (SE)	64.8 (2.5)	69.7 (3.4)	-	T = −1.16, *p* = 0.25
Diagnosis, % schizophrenia *	64.7	60.7	-	χ^2^ = 0.05, *df* = 1, *p* = 0.83
Age of onset, mean (SE)	19.3 (1.1)	19.6 (1.4)	-	T = −0.15, *p* = 0.88
Duration of illness, mean (SE)	14.7 (0.7)	14.4 (0.9)	-	T = 0.13, *p* = 0.89
Clozapine, % patients	38.2%	57.1%	-	χ^2^ = 2.035, *df* = 1, *p* = 0.154
Chlorpromazine equivalents (mg/day), mean (SE)	652.5 (86.8)	492.4 (60.4)	-	T = 1.51, *p* = 0.14
Antisocial personality trait score, mean (SE)	31.7 (1.3)	40.5 (2.0)	-	T = −3.7, *p* < 0.001

Abbreviations: Sch-V: nonviolent patients with schizophrenia; Sch+V: violent patients with schizophrenia; HC: healthy controls; SE = standard error. * Patients were either diagnosed with schizophrenia or schizoaffective disorder.

**Table 2:** Between-group differences in gray matter volume for sub-cortical regions of interest.

	All Groups			HC vs. Sch-V		HC vs. Sch+V		Sch-V vs. Sch+V	
Regions of Interest	F	Partial η^2^	FDR-Corrected *p*	t	FDR-Corrected *p*	t	FDR-Corrected *p*	t	FDR-Corrected *p*
Left Amygdala	3.66	0.09	0.08						
Right Amygdala	10.63	0.21	0.0003	1.81	0.07	4.12	0.0003	2.74	0.01
Left Accumbens	0.73	0.02	0.81						
Right Accumbens	3.13	0.07	0.10						
Left Caudate	0.09	0.002	0.91						
Right Caudate	0.15	0.004	0.91						
Left Putamen	0.25	0.006	0.91						
Right Putamen	0.11	0.003	0.91						

Abbreviations: Sch-V: nonviolent patients with schizophrenia; Sch+V: violent patients with schizophrenia; HC: healthy control.

**Table 3:** Between-group differences in cortical thickness for cortical regions of interest.

	All Groups			HC vs. Sch-V		HC vs. Sch+V		Sch-V vs. Sch+V	
Regions of Interest	F	Partial η^2^	FDR-Corrected *p*	t	FDR-Corrected *p*	t	FDR-Corrected *p*	t	FDR-Corrected *p*
Medial orbitofrontal	4.96	0.12	0.03	3.07	0.009	2.39	0.03	−0.65	0.52
Left lateral orbitofrontal	3.62	0.09	0.07						
Right lateral orbitofrontal	2.19	0.05	0.12						
Left pars orbitalis of the inferior frontal gyrus	3.52	0.08	0.07						
Right pars orbitalis of the inferior frontal gyrus	2.32	0.06	0.12						
Left caudal middle frontal	5.94	0.14	0.02	1.10	0.27	3.11	0.008	2.35	0.03
Right caudal middle frontal	8.26	0.18	0.006	1.89	0.06	3.81	0.0008	2.29	0.04
Caudal anterior cingulate	2.39	0.06	0.12						
Left insula	2.77	0.07	0.10						
Right insula	3.15	0.08	0.08						

Abbreviations: Sch-V: nonviolent patients with schizophrenia; Sch+V: violent patients with schizophrenia; HC: healthy controls.

## Data Availability

The data that support the findings of this study are available upon reasonable request from the corresponding author, S.P., but are only redistributable to researchers engaged in IRB-approved research collaborations. The data are not publicly available due to Ethical reasons.

## Abbreviations

The following abbreviations are used in this manuscript:

ACC	Anterior cingulate cortex
CT	Cortical thickness
dlPFC	Dorsolateral prefrontal cortex
FDR	False discovery rate
fMRI	Functional magnetic resonance imaging
GMV	Gray matter volume
HC	Healthy controls
MACVI	MacArthur Community Violence Instrument
MANCOVA	Multivariate analysis of covariance
OFC	Orbitofrontal cortex
PANSS	Positive and Negative Syndrome Scale
ROI	Region of interest
Sch	Schizophrenia
sMRI	Structural magnetic resonance imaging
TIV	Total intracranial volume
vlPFC	Ventrolateral prefrontal cortex

## References

[1] Swanson J.W., Swartz M.S., Van Dorn R.A., Elbogen E.B., Wagner H.R., Rosenheck R.A., Stroup T.S., McEvoy J.P., Lieberman J.A. (2006). A National Study of Violent Behavior in Persons with Schizophrenia. Arch. Gen. Psychiatry.

[2] Silverstein S.M., Del Pozzo J., Roché M., Boyle D., Miskimen T. (2015). Schizophrenia and Violence: Realities and Recommendations. Crime Psychol. Rev..

[3] Fazel S., Gulati G., Linsell L., Geddes J.R., Grann M. (2009). Schizophrenia and Violence: Systematic Review and Meta-Analysis. PLoS Med..

[4] Witt K., van Dorn R., Fazel S. (2013). Risk Factors for Violence in Psychosis: Systematic Review and Meta-Regression Analysis of 110 Studies. PLoS ONE.

[5] Witt K., Lichtenstein P., Fazel S. (2015). Improving Risk Assessment in Schizophrenia: Epidemiological Investigation of Criminal History Factors. Br. J. Psychiatry.

[6] Hachtel H., Nixon M., Bennett D., Mullen P., Ogloff J. (2018). Motives, Offending Behavior, and Gender Differences in Murder Perpetrators With or Without Psychosis. J. Interpers. Violence.

[7] Reinharth J., Reynolds G., Dill C., Serper M. (2014). Cognitive Predictors of Violence in Schizophrenia: A Meta-Analytic Review. Schizophr. Res. Cogn..

[8] Hodgins S., Riaz M. (2011). Violence and Phases of Illness: Differential Risk and Predictors. Eur. Psychiatry.

[9] Bulgari V., Iozzino L., Ferrari C., Picchioni M., Candini V., De Francesco A., Maggi P., Segalini B., de Girolamo G. (2017). Clinical and Neuropsychological Features of Violence in Schizophrenia: A Prospective Cohort Study. Schizophr. Res..

[10] Buchanan A., Sint K., Swanson J., Rosenheck R. (2019). Correlates of Future Violence in People Being Treated for Schizophrenia. Am. J. Psychiatry.

[11] Hodgins S. (2017). Aggressive Behavior Among Persons with Schizophrenia and Those Who Are Developing Schizophrenia: Attempting to Understand the Limited Evidence on Causality. Schizophr. Bull..

[12] World Health Organization Schizophrenia. https://www.who.int/news-room/fact-sheets/detail/schizophrenia.

[13] Hodgins S., Janson C.-G. (2009). Criminality and Violence Among the Mentally Disordered: The Stockholm Metropolitan Project.

[14] Hodgins S. (2008). Violent Behaviour among People with Schizophrenia: A Framework for Investigations of Causes, and Effective Treatment, and Prevention. Philos. Trans. R. Soc. Lond. B Biol. Sci..

[15] Fazel S., Wolf A., Fimińska Z., Larsson H. (2016). Mortality, Rehospitalisation and Violent Crime in Forensic Psychiatric Patients Discharged from Hospital: Rates and Risk Factors. PLoS ONE.

[16] Torrey E.F. (2011). Stigma and Violence: Isn’t It Time to Connect the Dots?. Schizophr. Bull..

[17] Volavka J., Citrome L. (2008). Heterogeneity of Violence in Schizophrenia and Implications for Long-Term Treatment. Int. J. Clin. Pract..

[18] Nikolic M., Pezzoli P., Jaworska N., Seto M.C. (2022). Brain Responses in Aggression-Prone Individuals: A Systematic Review and Meta-Analysis of Functional Magnetic Resonance Imaging (FMRI) Studies of Anger- and Aggression-Eliciting Tasks. Prog. Neuropsychopharmacol. Biol. Psychiatry.

[19] Dugré J.R., Radua J., Carignan-Allard M., Dumais A., Rubia K., Potvin S. (2020). Neurofunctional Abnormalities in Antisocial Spectrum: A Meta-Analysis of FMRI Studies on Five Distinct Neurocognitive Research Domains. Neurosci. Biobehav. Rev..

[20] Raschle N.M., Menks W.M., Fehlbaum L.V., Tshomba E., Stadler C. (2015). Structural and Functional Alterations in Right Dorsomedial Prefrontal and Left Insular Cortex Co-Localize in Adolescents with Aggressive Behaviour: An ALE Meta-Analysis. PLoS ONE.

[21] Aoki Y., Inokuchi R., Nakao T., Yamasue H. (2014). Neural Bases of Antisocial Behavior: A Voxel-Based Meta-Analysis. Soc. Cogn. Affect. Neurosci..

[22] Dugré J.R., De Brito S.A. (2024). Unraveling the Morphological Brain Architecture of Human Aggression: A Systematic Review and Meta-Analysis of Structural Neuroimaging Studies. Aggress. Violent Behav..

[23] De Brito S.A., McDonald D., Camilleri J.A., Rogers J.C. (2021). Cortical and Subcortical Gray Matter Volume in Psychopathy: A Voxel-Wise Meta-Analysis. J. Abnorm. Psychol..

[24] Bobes M.A., Ostrosky F., Diaz K., Romero C., Borja K., Santos Y., Valdés-Sosa M. (2013). Linkage of Functional and Structural Anomalies in the Left Amygdala of Reactive-Aggressive Men. Soc. Cogn. Affect. Neurosci..

[25] Joyal C.C., Putkonen A., Mancini-Marïe A., Hodgins S., Kononen M., Boulay L., Pihlajamaki M., Soininen H., Stip E., Tiihonen J. (2007). Violent Persons with Schizophrenia and Comorbid Disorders: A Functional Magnetic Resonance Imaging Study. Schizophr. Res..

[26] Kumari V., Aasen I., Taylor P., Ffytche D.H., Das M., Barkataki I., Goswami S., O’Connell P., Howlett M., Williams S.C.R. (2006). Neural Dysfunction and Violence in Schizophrenia: An FMRI Investigation. Schizophr. Res..

[27] Tikàsz A., Potvin S., Richard-Devantoy S., Lipp O., Hodgins S., Lalonde P., Lungu O., Dumais A. (2018). Reduced Dorsolateral Prefrontal Cortex Activation during Affective Go/NoGo in Violent Schizophrenia Patients: An FMRI Study. Schizophr. Res..

[28] Tikàsz A., Potvin S., Lungu O., Joyal C.C., Hodgins S., Mendrek A., Dumais A. (2016). Anterior Cingulate Hyperactivations during Negative Emotion Processing among Men with Schizophrenia and a History of Violent Behavior. Neuropsychiatr. Dis. Treat..

[29] Widmayer S., Borgwardt S., Lang U.E., Stieglitz R.D., Huber C.G. (2019). Functional Neuroimaging Correlates of Aggression in Psychosis: A Systematic Review with Recommendations for Future Research. Front. Psychiatry.

[30] Wang Y., Zhang Y., Wang Y., Cao Q., Zhang M. (2024). Task-Related Brain Activation Associated with Violence in Patients with Schizophrenia: A Meta-Analysis. Asian J. Psychiatr..

[31] Athanassiou M., Dumais A., Tikasz A., Lipp O., Dubreucq J.L., Potvin S. (2022). Increased Cingulo-Orbital Connectivity Is Associated with Violent Behaviours in Schizophrenia. J. Psychiatr. Res..

[32] Tikàsz A., Potvin S., Dugré J.R., Fahim C., Zaharieva V., Lipp O., Mendrek A., Dumais A. (2020). Violent Behavior Is Associated With Emotion Salience Network Dysconnectivity in Schizophrenia. Front. Psychiatry.

[33] Hoptman M.J., D’Angelo D., Catalano D., Mauro C.J., Shehzad Z.E., Kelly A.M.C., Castellanos F.X., Javitt D.C., Milham M.P. (2010). Amygdalofrontal Functional Disconnectivity and Aggression in Schizophrenia. Schizophr. Bull..

[34] Kumari V., Uddin S., Premkumar P., Young S., Gudjonsson G.H., Raghuvanshi S., Barkataki I., Sumich A., Taylor P., Das M. (2014). Lower Anterior Cingulate Volume in Seriously Violent Men with Antisocial Personality Disorder or Schizophrenia and a History of Childhood Abuse. Aust. N. Z. J. Psychiatry.

[35] Kuroki N., Kashiwagi H., Ota M., Ishikawa M., Kunugi H., Sato N., Hirabayashi N., Ota T. (2017). Brain Structure Differences among Male Schizophrenic Patients with History of Serious Violent Acts: An MRI Voxel-Based Morphometric Study. BMC Psychiatry.

[36] Kolla N.J., Harenski C.L., Harenski K.A., Dupuis M., Crawford J.J., Kiehl K.A. (2021). Brain Gray Matter Differences among Forensic Psychiatric Patients with Psychosis and Incarcerated Individuals without Psychosis: A Source-Based Morphometry Study. Neuroimage Clin..

[37] Kumari V., Gudjonsson G.H., Raghuvanshi S., Barkataki I., Taylor P., Sumich A., Das K., Kuipers E., Ffytche D.H., Das M. (2013). Reduced Thalamic Volume in Men with Antisocial Personality Disorder or Schizophrenia and a History of Serious Violence and Childhood Abuse. Eur. Psychiatry.

[38] Hoptman M.J., Volavka J., Weiss E.M., Czobor P., Szeszko P.R., Gerig G., Chakos M., Blocher J., Citrome L.L., Lindenmayer J.P. (2005). Quantitative MRI Measures of Orbitofrontal Cortex in Patients with Chronic Schizophrenia or Schizoaffective Disorder. Psychiatry Res..

[39] Storvestre G.B., Valnes L.M., Jensen A., Nerland S., Tesli N., Hymer K.E., Rosaeg C., Server A., Ringen P.A., Jacobsen M. (2019). A Preliminary Study of Cortical Morphology in Schizophrenia Patients with a History of Violence. Psychiatry Res. Neuroimaging.

[40] Fjellvang M., Grøning L., Haukvik U.K. (2018). Imaging Violence in Schizophrenia: A Systematic Review and Critical Discussion of the MRI Literature. Front. Psychiatry.

[41] Leclerc M.P., Regenbogen C., Hamilton R.H., Habel U. (2018). Some Neuroanatomical Insights to Impulsive Aggression in Schizophrenia. Schizophr. Res..

[42] Wang Y., Wang Y., Cao Q., Zhang M. (2023). Aberrant Brain Structure in Patients with Schizophrenia and Violence: A Meta-Analysis. J. Psychiatr. Res..

[43] Widmayer S., Sowislo J.F., Jungfer H.A., Borgwardt S., Lang U.E., Stieglitz R.D., Huber C.G. (2018). Structural Magnetic Resonance Imaging Correlates of Aggression in Psychosis: A Systematic Review and Effect Size Analysis. Front. Psychiatry.

[44] Lamsma J., Raine A., Kia S.M., Cahn W., Arold D., Banaj N., Barone A., Brosch K., Brouwer R., Brunetti A. (2024). Structural Brain Abnormalities and Aggressive Behaviour in Schizophrenia: Mega-Analysis of Data from 2095 Patients and 2861 Healthy Controls via the ENIGMA Consortium. medRxiv.

[45] Kay S.R., Fiszbein A., Opler L.A. (1987). The Positive and Negative Syndrome Scale (PANSS) for Schizophrenia. Schizophr. Bull..

[46] American Psychiatric Association (2013). Diagnostic and Statistical Manual of Mental Disorders.

[47] Steadman H., Silver E. (1999). Immediate Precursors of Violence among Persons with Mental Illness: A Return to a Situational Perspective. Violence Among the Mentally Ill.

[48] Monahan J., Steadman H.J., Silver E., Appelbaum P.S., Robbins P.C., Mulvey E.P., Roth L.H., Grisso T., Banks S. (2001). Rethinking Risk Assessment: The MacArthur Study of Mental Disorder and Violence.

[49] Appelbaum P.S., Robbins P.C., Monahan J. (2000). Violence and Delusions: Data from the MacArthur Violence Risk Assessment Study. Am. J. Psychiatry.

[50] Cartwright J.K., Desmarais S.L., Grimm K.J., Meade A.W., Van Dorn R.A. (2020). Psychometric Properties of the MacArthur Community Violence Screening Instrument. Int. J. Forensic Ment. Health.

[51] Bell C., Tesli N., Gurholt T.P., Rokicki J., Hjell G., Fischer-Vieler T., Melle I., Agartz I., Andreassen O.A., Rasmussen K. (2022). Associations between Amygdala Nuclei Volumes, Psychosis, Psychopathy, and Violent Offending. Psychiatry Res. Neuroimaging.

[52] First M., Gibbon M., Hilsenroth M., Segal J. (2004). The Structured Clinical Interview for DSM-IV Axis I Disorders (SCID-I) and the Structured Clinical Interview for DSM-IV Axis II Disorders (SCID-II). Comprehensive Handbook of Psychological Assessment, Personality Assessment.

[53] Leucht S., Samara M., Heres S., Davis J.M. (2016). Dose Equivalents for Antipsychotic Drugs: The DDD Method. Schizophr. Bull..

[54] Fischl B. (2012). FreeSurfer. Neuroimage.

[55] Schumacker R.E. (2016). Using R with Multivariate Statistics.

[56] Glickman M.E., Rao S.R., Schultz M.R. (2014). False Discovery Rate Control Is a Recommended Alternative to Bonferroni-Type Adjustments in Health Studies. J. Clin. Epidemiol..

[57] Benjamini Y., Hochberg Y. (1995). Controlling the False Discovery Rate: A Practical and Powerful Approach to Multiple Testing. J. R. Stat. Soc. Ser. B.

[58] Li Y., Liang W., Zhao L. (2024). Quantitative Gray Matter Volumetric Analysis in Schizophrenia: Investigating the Risk of Violent Behaviors through Structural MRI. Egypt. J. Radiol. Nucl. Med..

[59] Coccaro E.F., Sripada C.S., Yanowitch R.N., Phan K.L. (2011). Corticolimbic Function in Impulsive Aggressive Behavior. Biol. Psychiatry.

[60] Davidson R.J., Putnam K.M., Larson C.L. (2000). Dysfunction in the Neural Circuitry of Emotion Regulation—A Possible Prelude to Violence. Science.

[61] Fanning J.R., Keedy S., Berman M.E., Lee R., Coccaro E.F. (2017). Neural Correlates of Aggressive Behavior in Real Time: A Review of FMRI Studies of Laboratory Reactive Aggression. Curr. Behav. Neurosci. Rep..

[62] Achterberg M., van Duijvenvoorde A.C.K., Bakermans-Kranenburg M.J., Crone E.A. (2016). Control Your Anger! The Neural Basis of Aggression Regulation in Response to Negative Social Feedback. Soc. Cogn. Affect. Neurosci..

[63] Achterberg M., van Duijvenvoorde A.C.K., van IJzendoorn M.H., Bakermans-Kranenburg M.J., Crone E.A. (2020). Longitudinal Changes in DLPFC Activation during Childhood Are Related to Decreased Aggression Following Social Rejection. Proc. Natl. Acad. Sci. USA.

[64] Chester D.S., DeWall C.N. (2016). The Pleasure of Revenge: Retaliatory Aggression Arises from a Neural Imbalance toward Reward. Soc. Cogn. Affect. Neurosci..

[65] Roberton T., Daffern M., Bucks R.S. (2012). Emotion Regulation and Aggression. Aggress. Violent Behav..

[66] Dolcos F., Iordan A.D., Dolcos S. (2011). Neural Correlates of Emotion–Cognition Interactions: A Review of Evidence from Brain Imaging Investigations. J. Cogn. Psychol..

[67] Barkataki I., Kumari V., Das M., Taylor P., Sharma T. (2006). Volumetric Structural Brain Abnormalities in Men with Schizophrenia or Antisocial Personality Disorder. Behav. Brain Res..

[68] Tesli N., van der Meer D., Rokicki J., Storvestre G., Røsæg C., Jensen A., Hjell G., Bell C., Fischer-Vieler T., Tesli M. (2020). Hippocampal Subfield and Amygdala Nuclei Volumes in Schizophrenia Patients with a History of Violence. Eur. Arch. Psychiatry Clin. Neurosci..

[69] Berboth S., Morawetz C. (2021). Amygdala-Prefrontal Connectivity during Emotion Regulation: A Meta-Analysis of Psychophysiological Interactions. Neuropsychologia.

[70] Ocklenburg S., Peterburs J., Mundorf A. (2022). Hemispheric Asymmetries in the Amygdala: A Comparative Primer. Prog. Neurobiol..

[71] Baas D., Aleman A., Kahn R.S. (2004). Lateralization of Amygdala Activation: A Systematic Review of Functional Neuroimaging Studies. Brain Res. Rev..

[72] Yoshida N., Kotani Y., Ohgami Y., Kunimatsu A., Inoue Y., Kiryu S., Okada Y. (2021). Effects of Negativity Bias on Amygdala and Anterior Cingulate Cortex Activity in Short and Long Emotional Stimulation Paradigms. Neuroreport.

[73] Gläscher J., Adolphs R. (2003). Processing of the Arousal of Subliminal and Supraliminal Emotional Stimuli by the Human Amygdala. J. Neurosci..

[74] Lickley R.A., Sebastian C.L. (2018). The Neural Basis of Reactive Aggression and Its Development in Adolescence. Psychol. Crime Law.

[75] Coccaro E.F., Lee R., McCloskey M., Csernansky J.G., Wang L. (2015). Morphometric Analysis of Amygdla and Hippocampus Shape in Impulsively Aggressive and Healthy Control Subjects. J. Psychiatr. Res..

[76] Coccaro E.F., Fitzgerald D.A., Lee R., McCloskey M., Phan K.L. (2016). Frontolimbic Morphometric Abnormalities in Intermittent Explosive Disorder and Aggression. Biol. Psychiatry Cogn. Neurosci. Neuroimaging.

[77] O’Reilly K., O’Connell P., O’Sullivan D., Corvin A., Sheerin J., O’Flynn P., Donohoe G., McCarthy H., Ambrosh D., O’Donnell M. (2019). Moral Cognition, the Missing Link between Psychotic Symptoms and Acts of Violence: A Cross-Sectional National Forensic Cohort Study. BMC Psychiatry.

[78] O’Reilly K., Donohoe G., Coyle C., O’Sullivan D., Rowe A., Losty M., McDonagh T., McGuinness L., Ennis Y., Watts E. (2015). Prospective Cohort Study of the Relationship between Neuro-Cognition, Social Cognition and Violence in Forensic Patients with Schizophrenia and Schizoaffective Disorder. BMC Psychiatry.

[79] Kolla N.J., Houle S. (2019). Single-Photon Emission Computed Tomography and Positron Emission Tomography Studies of Antisocial Personality Disorder and Aggression: A Targeted Review. Curr. Psychiatry Rep..

[80] Abbott C.C., Jaramillo A., Wilcox C.E., Hamilton D.A. (2013). Antipsychotic Drug Effects in Schizophrenia: A Review of Longitudinal FMRI Investigations and Neural Interpretations. Curr. Med. Chem..

[81] Gonzalez-Vivas C., Soldevila-Matias P., Sparano O., Garcia-Marti G., Marti-Bonmati L., Crespo-Facorro B., Aleman A., Sanjuan J. (2019). Longitudinal Studies of Functional Magnetic Resonance Imaging in First-Episode Psychosis: A Systematic Review. Eur. Psychiatry.

[82] Vita A., De Peri L., Deste G., Sacchetti E. (2012). Progressive Loss of Cortical Gray Matter in Schizophrenia: A Meta-Analysis and Meta-Regression of Longitudinal MRI Studies. Transl. Psychiatry.

[83] Mendrek A., Mancini-Marïe A. (2016). Sex/Gender Differences in the Brain and Cognition in Schizophrenia. Neurosci. Biobehav. Rev..

